# Physicochemical and antioxidant properties of pectin from *Actinidia arguta* Sieb.et Zucc (*A. arguta*) extracted by ultrasonic

**DOI:** 10.3389/fnut.2024.1349162

**Published:** 2024-04-10

**Authors:** Liqi Liu, Yuhan Sui, Tienan Wang, Xiang Li, Lina Chen, Mao Shi

**Affiliations:** ^1^College of Food Science and Engineering, Changchun University, Changchun, China; ^2^Jilin Provincial Center for Disease Control and Prevention, Changchun, China

**Keywords:** *Actinidia arguta* Sieb. et Zucc (*A.arguta*), pectin, ultrasonic, physical properties, chemical properties, oxidize resistance

## Abstract

Pectin was extracted from *Actinidia arguta* Sieb. et Zucc (*A.arguta*) using the ultrasound-assisted acid method and the single acid method. The physicochemical properties, structure, and antioxidant properties of two different pectins were investigated. The results showed that the extraction yield of the ultrasound-assisted acid method is higher than that of the single acid method. The molecular structure of *A. arguta* pectin extracted by the ultrasound-assisted acid method belongs to a mixed structure of RG-I and HG-type domains. Through structural feature analysis, the ultrasound-assisted extraction pectin (UAP) has a more branched structure than the single acid-extracted pectin (SAP). The SAP has a higher degree of esterification than the UAP. The physical property results show that the viscosity, solubility, and water-holding capacity of the UAP are better than those of the SAP. The antioxidant test results show that the hydroxyl radical scavenging and reducing powers of the UAP are superior to those of the SAP. This study shows the composition, physicochemical properties, and antioxidant activity of *A. arguta* pectin extracted by the ultrasonic-assisted extraction method to provide a theoretical basis for its application as an antioxidant and other food additives in the food industry.

## 1 Introduction

Pectin is a natural, high-molecular-weight polysaccharide derived from plants, belonging to the acidic heteropolysaccharide family. It is widely present in plants, especially fruits and vegetables, and is the main component of plant cell walls and inner layers (1). The chemical composition and structure of pectin vary with plant species, growth environments, seasons, and processing techniques. Its essence is a linear structure of polysaccharides, mainly composed of D-galactonic acid. D-Galacturonic acid is composed of α-1,4 glycosidic bonds to form slightly acidic macromolecular polysaccharides (2). The molecular main chain and branch chain structures of pectin are different. They are divided into four configurations, namely, homocysteine, rhamnogalacturonan I, rhamnogalacturonan II, and xylogalacturonan (3). The backbone of RG-I is different: it is composed of repeating units of galacturonic acid (GalA) residues and rhamnose (Rha). RG-I has numerous side chains, which are mainly linked to the O-4 rhamnose units and contain neutral sugars like arabinans, galactans, and/or arabinogalactans (4). HG is the simplest pectin, and therefore its functionality is best understood due to experimental and theoretical studies. The HG backbone is relatively stiff and may change helical conformation due to the degree of methylation. In contrast to HG, the RG-I sections are more flexible, creating the so-called “hairy” regions of pectin. Moreover, neutral sugars are linked to the RG-I backbone at the rhamnose unit (5). A significantly more complicated structure and variability due to plant species and the physiological state of the plant or extraction method have resulted in RG-I being less well-understood. In the past few years, people have begun to pay attention to the health properties of pectin. Studies have shown that the configuration is an important parameter of pectin, which is closely related to the physical and chemical properties and biological activity of pectin (1). Studies have shown that substituting rhamnose-galacturonic acid (RGs) for pectin can prevent cancer (4) by inducing cancer cell apoptosis (3).

There are many methods for extracting pectin, including acid extraction (1), alkali extraction (1), ion exchange (5), microwave extraction (6), ultrasonic extraction (7), enzyme extraction (8), continuous countercurrent extraction (9), and supercritical fluid extraction (10). In industrial production, acid extraction is often used to extract pectin from fruits such as apples and citrus. Ultrasonic extraction utilizes the mechanical effects, cavitation effects, and thermal effects of ultrasound to extract bioactive components by increasing the movement speed of medium molecules and the penetration force of the medium (11). The unique physical characteristics of ultrasound can promote the breaking or deformation of plant cell tissues, making the extraction of effective ingredients more sufficient. The extraction rate is significantly increased by 50–500% compared to traditional processes, and the raw materials are not limited by component polarity or molecular weight size (12). It is suitable for the extraction of the vast majority of various components in raw materials, with fewer impurities in the extraction solution, easy separation and purification of effective ingredients, low operating costs of the extraction process, and significant comprehensive economic benefits.

As a source of pectin, fruit processing by-products have enormous value-added potential. Pectin solution has a high viscosity, and its gel properties can be used in the production of various jellies, jams, and other foods, giving people a unique flavor; it can also be used as a thickener and stabilizer for juice, candy products, and dairy products (13). According to the National Food Safety Standard for the Use of Food Additives People's GB/T 2760-86 (Republic of China National Standard), pectin can be used as a thickener, stabilizer, emulsifier, gelling agent, and so on. It can be added to yogurt products, canned goods, jam, fruit juice, and other foods without daily dosage restrictions.

*Actinidia arguta* Sieb. et Zucc (*A.arguta*), also known as *kiwi berry, soft jujube fruit*, and so on, is a deciduous vine plant of the *kiwifruit family* and *genus Kiwifruit* in foreign countries (14). It is a precious cold-resistant fruit tree resource in China (15). *A. arguta* has a high nutritional value and contains rich bioactive substances such as pectin polysaccharides, alkaloids, superoxide dismutase (SOD), volatile oils, and flavonoids (16). It is precisely because of the presence of these bioactive substances that *A. arguta* has medicinal values such as antioxidant (17), anti-inflammatory, anti-tumor, hypoglycemic, lipid-lowering, and anti-fatigue (18). At present, research on the technology of *A. arguta* in China focuses on variety selection, cultivation techniques, and component analysis such as vitamin C and polyphenols (19), but there is not much research on pectin and processing techniques (20). Pectin, as an important component of *A. arguta*, has a content of about 15–20% and has physiological functions such as anticancer and cholesterol reduction for the human body. It can be used as an emulsification stabilizer to improve and stabilize the taste (21). Based on the biological activity of *A. arguta* pectin, clarifying its composition information can provide theoretical guidance for the development of functional products of *A. arguta*.

As a unique resource in Northeast China, *A. arguta* dates have been expanding with the artificial planting area in recent years and entering a large number of fruit ripening periods (22). Due to its seasonality and poor storage resistance, deep processing will avoid the impact of concentrated ripening on fruit prices and resource waste and fill the gap in deep processing technology and products for *A. arguta* dates (23). By developing and utilizing the by-products of *A. arguta* processing, fully preserving their nutritional components, and developing food additives, not only can the comprehensive utilization rate of *A. arguta* resources and product-added value be improved, but it also has very important social significance for promoting the rapid and healthy development of the industry and promoting the local economic development of Jilin Province.

Previous studies mainly focused on the preparation process, physicochemical properties, and pharmacological effects of pectin, while this paper mainly studied and compared the composition, physicochemical properties, and antioxidant activities of pectin prepared by the ultrasonic method to provide a theoretical basis for its application as an antioxidant in food processing.

## 2 Materials and methods

### 2.1 Materials and reagents

*A. arguta* (Ultramarine-green) was provided by the College of Landscape Architecture, Changchun University; carbazole (≥99%), potassium bromide (≥99%), salicylic acid (≥99%), coomassie brilliant blue (≥99%), petroleum ether (≥99%), sodium hydroxide (≥99%), and so on are purchased from Beijing Dingguo Biology (Beijing, China); standard monosaccharides are purchased from Sigma-Aldrich (Shanghai) Trading Co., Ltd (Shanghai, China).

### 2.2 Ultrasonic-assisted extraction of *A. arguta* pectin

The *A. arguta* was dried in the oven at 80°C and then crushed by the ultrafine grinder through the 80-mesh screen to obtain *A. arguta* powder. *A. arguta* powder was dissolved in deionized water according to the ratio of solid to liquid 1:110 (g/ml) by adjusting pH with hydrochloric acid to 2.3 and extracted in a water bath at 80°C for 2 h, and the acid-extracted pectin (SAP) was prepared. The ultrasound-assisted extraction pectin (UAP) was used to dissolve *A. arguta* powder with deionized water according to the ratio of solid to liquid (1:110 g/ml) by adjusting pH with hydrochloric acid to 2.3 and treated by ultrasonic cell crusher (BILON-1000Y, Shanghai Bilang Instrument Manufacturing Co., Ltd). The ultrasonic power is 200 W, the ultrasonic reactor frequency is 15 s, and the ultrasonic time is 10 min (24, 25). Then it was extracted in an 80°C water bath for 2 h. The pectin extracts prepared by the two methods were centrifuged at 4,000 r/min for 20 min. The supernatant was taken, concentrated by a rotary evaporator (RE-52AA, Shanghai Yarong Biochemical Instrument Factory), treated with anhydrous ethanol for 12 h, and precipitated at 4,000 r/min for 20 min. After freeze-drying (VACO5-80, ZIRBUS, Germany), the *A. arguta* pectin powder was obtained.

The content of D-galacturonic acid was determined by the carbazol-sulfuric acid method to indicate the content of pectin (26). With galacturonic acid as the standard, a standard curve was drawn using the mass concentration of galacturonic acid as the x-axis and absorbance as the y-axis (27). Dilute the pectin extract to a certain mass concentration, follow the standard curve steps, and calculate the pectin yield according to the formula (1).


(1) $$\begin{array}{l} \text{Pectin yield}\%=\frac{\text{C}\times \text{V}\times \text{N}}{\text{M}}\times 100 \end{array}$$

C is the mass concentration of galacturonic acid in the solution/μg/ml; V is the total volume/ml of pectin extract; N is the dilution ratio of the extraction solution; and M is the mass of *A. arguta* power (28).

### 2.3 Chemical composition of *A. arguta* pectin

For the sample chemical composition index, total galacturonic acid, total sugar, total protein, moisture, and ash of the sample were determined. The determination of total galacturonic acid content in pectin refers to the alkali titration (29). The determination of total sugar content is carried out using the phenol sulfuric acid method, in which glucose was used as a standard (30). Protein content determination was determined using the Kjeldahl method (31). Moisture and ash content refer to the weight loss method (32). The pH value was measured at room temperature by a digital pH detector (pHS-3C, Shanghai Yi Electrical Scientific Instruments Co., Ltd). The degree of esterification of pectin was calculated by acid-base titration (1).

### 2.4 Monosaccharide composition

The monosaccharide composition analysis used arabinose, xylose, galactose, mannose, galacturonic acid, glucuronic acid, rhamnose, glucose, and fructose as monosaccharide standards. Monosaccharide composition using high-performance liquid chromatography (HPLC, LC-20AT), SPD-10AVD ultraviolet detector, DIKMA Inertsil ODS-3 chromatographic column (4.6 × 150 mm), mobile phase is a mixture of PBS (0.1 mol/L, pH 7.0) and acetonitrile in a volume ratio of 82:18, flow rate of 1.0 ml/min, and detection wavelength of 245 nm. An amount of 1 mg of sample was weighed, 1 ml of hydrochloric acid methanol solution was added into an acid hydrolysis vial, N_2_ was filled, and the vial was allowed to react in a metal bath at 80°C for 16 h. After the reaction, the sample was dried with methanol hydrochloric acid by a nitrogen blower, and 1 ml of 2 M trifluoroacetic acid (TFA) was added to the sample at 120°C for 1 h. The sample was blow dried after the reaction is over. A volume of 500 μl of 0.3 M NaOH was added to the acid hydrolysis vial to dissolve the monosaccharide sample completely. A volume of 500 μl of 0.5 M PMP-methanol was added, and 200 μl of the mixed solution was taken into the EP tube and put into a 70°C water bath for 30 min. Then, 100 μl of 0.3 M HCl was added and extracted. A volume of 700 μl of dichloromethane was added. After centrifuging, shaking, and pumping away the lower organic phase, EP was filtered through a 0.22 μm organic filter membrane, leaving the remaining aqueous phase intact. The samples were prepared using HPLC (33).

### 2.5 Molecular weight

Determination of relative molecular weight: A quantity of 1 mg of extracted pectin was dissolved in a 0.2 M NaCl solution and filtered with a 0.22 μm membrane. The molecular weight distribution was measured and analyzed using LC-10Avp high-performance liquid chromatography. Instrument: waters ultrahydrogel linear, 7.8 × 300 mm; differential refraction detector; column temperature, 30°C; flow rate, 0.5 ml/min; sample volume, 50 pl; mobile phase; pH = 7.0 phosphate buffer (34).

### 2.6 Fourier transform infrared spectroscopy

An infrared spectrometer (Thermo Nicolet NEXUS 670, United States) was used to analyze the infrared spectroscopy characterization. The samples of pectin were freeze-dried, smashed into a powder, and mixed with potassium bromide (1:200). The infrared spectrum of the prepared samples was recorded by FT-IR between 4,500 and 500 cm^−1^ (29).

### 2.7 Scanning electron microscopy

A scanning electron microscope (SEM; JEOLJSM-7500, Oberkochen, Germany) was used to observe and take photographs of the surface microstructure of pectin under different extraction methods. An appropriate amount of *A. arguta* pectin powder was taken, the dried *A. arguta* pectin was evenly distributed on the sample stage, a thin layer of gold was sputtered under vacuum conditions, SEM images were collected using a scanning electron microscope at an accelerating voltage of 15.0 kV, and the images were amplified by 500, 1,000, and 2,000 times (35).

### 2.8 Physical properties of *A. arguta* pectin

The viscosity of pectin samples was determined using a rheometer (RSO, Brookfield, USA). Rpo-25 was selected for the measurement system, and the shear rate was selected for the mode. The measurement conditions were temperature (25°C), shear rate (120.000 s^−1^), residence time (10 s), and duration of measuring point (10 s). Parallel measurement was performed three times, and the average value was taken (36).

The hardness was measured using a texture analyzer (EZ-SX, Shimadzu Corporation, Japan). The test conditions are P/50 probe, trigger force 0.05 N automatic trigger type, probe immersion distance is set to 5 mm, and two compression pause time is 3 s. The speed during the test and the speed when the probe is returned after the test are set to 1.0 mm/s. With the maximum breaking force as the hardness, each treatment was repeated three times, and the results were averaged (37).

#### 2.8.1 Determination of solubility

A quantity of 20 mg of *A. arguta* pectin (m_2_) in a beaker was weighed; 50 ml of pure water was added to dissolve; the solution was centrifuged for 30 min; and the supernatant was taken, placed in a beaker with a known constant weight, and dried at 110°C to a constant weight (m_1_). The solubility of pectin was calculated according to the formula (2) (38).


(2) $$\begin{array}{l} \text{Solubility}\left(\text{\%}\right)\;=\;\frac{m_{1}}{m_{2}}\;\times \;100\% \end{array}$$

#### 2.8.2 Determination of water-holding capacity

A 50 ml centrifuge tube was weighed as M_1_; then the dry powder sample of 0.5 g (M_2_) was weighed accurately and placed in the 50 ml centrifuge tube; 50 ml ultra-pure water was added, shaken well, centrifuged at a speed of 3,500 r/min for 15 min; the supernatant of the centrifuge liquid was discarded; and the total weight of the centrifuge tube containing sediment M_3_ was weighed. Then the sediment weight was calculated as (M_3_-M_1_). The water-holding capacity of pectin was calculated according to the formula (3) as follows (39).


(3) $$\begin{array}{l} \text{Water holding capacitiy}\left(\text{g}/\text{g}\right)\;=\;\frac{{\text{M}}_{3}\;-\;{\text{M}}_{1}\;-\;{\text{M}}_{2}}{M_{2}} \end{array}$$

### 2.9 Antioxidant activity of *A. arguta* pectin

#### 2.9.1 DPPH-free radical scavenging ability

The pectin solution of *A. arguta* was prepared with mass concentrations of 0.5, 1, 2, 5, and 10 mg/ml, respectively. Using Vc as a positive control, it was also configured according to the above concentration gradient, and then a certain volume of DPPH ethanol solution was added to the test tube under the conditions of room temperature and dark light, respectively. After the solution was mixed, the absorbance of the sample solution at the wavelength of 517 nm was measured by an ultraviolet spectrophotometer. Three parallel tests were conducted with anhydrous ethanol as the reference. The formula (4) is as follows.


(4) $$\begin{array}{l} \text{Clearance rate }\left(\text{\%}\right)\;=\;\left(1-\frac{A_{1}\;-\;A_{2}}{A_{0}}\right)\;\times \;100\% \end{array}$$

where A_1_ is the absorbance of 2 ml of kiwifruit pectin solution and 2 ml of DPPH. A_2_ is the absorbance of 2 ml of *A. arguta* pectin solution and 2 ml of anhydrous ethanol. A_0_ is the absorbance of 2 ml of dH_2_O+ 2 ml DPPH (40, 41).

#### 2.9.2 Hydroxyl radical scavenging ability

The pectin solution of *A. arguta* was prepared with mass concentrations of 0.5, 1, 2, 5, and 10 mg/ml, respectively. Using Vc as a positive control, it was also configured according to the above concentration gradient, and then the same volume of 6 mmol/L FeSO_4_ solution, 6 mmol/L H_2_O_2_ solution, and different mass concentrations of *A. arguta* pectin solution or Vc solution were mixed in the test tube. Then, the same volume of 6 mmol/L salicylic acid-ethanol solution was added to the water bath at 37°C for 1 h. After taking it out and cooling it to room temperature, the absorbance of the sample solution at the wavelength of 510 nm was measured by an ultraviolet spectrophotometer. Three parallel tests were carried out with distilled water as the reference. The formula (5) is as follows (40, 41).


(5) $$\begin{array}{l} \text{Clearance rate }\left(\text{\%}\right)\;=\;\left(1-\frac{A_{1}\;-\;A_{2}}{A_{0}}\right)\;\times \;100\% \end{array}$$

where *A*_1_ is the absorbance value of 2 ml FeSO_4_ solution + 2 ml H_2_O_2_ solution + 2 ml salicylic acid solution + 2 ml sample solution; *A*_2_ is the absorbance value of 2 ml FeSO_4_ solution + 2 ml dH_2_O+2 ml salicylic acid solution + 2 ml sample solution. *A*_0_ is the absorbance value of 2 ml FeSO_4_ solution + 2 ml H_2_O_2_ solution + 2 ml salicylic acid solution + 2 ml dH_2_O.

#### 2.9.3 Determination of ABTS-free radical clearance

The pectin solution of *A. arguta* was prepared with mass concentrations of 0.5, 1, 2, 5, and 10 mg/ml, respectively. Using Vc as a positive control, it was also configured according to the above concentration gradient. ABTS+ mother liquor was prepared from 200 mg of accurately weighed ABTS and 34.4 mg of potassium persulfate dissolved in 50 ml of distilled water and placed away from light at room temperature for 24 h. The absorbance was measured to (0.70 ± 0.02) at 734 nm by diluting the mother liquor with 95% ethanol. A volume of 0.4 ml of sample solution was added to 3.6 ml of diluted ABTS solution and allowed to react at 30°C for 5 min. Then, the absorbance A1 is determined at 734 nm, and the absorbance A0 is determined with distilled water instead of the sample solution. The calculation formula (6) is as follows (40, 41).


(6) $$\begin{array}{l} \text{ABTS free radical clearance rate }\left(\text{\%}\right)\;=\;\frac{A_{1}\;-\;A_{2}}{A_{0}}\;\times \;100\% \end{array}$$

#### 2.9.4 Determination of reducing power

The pectin solution of *A. arguta* was prepared with mass concentrations of 0.5, 1, 2, 5, and 10 mg/ml, respectively. Using Vc as a positive control, it was also configured according to the above concentration gradient. A volume of 1.0 ml of pectin solution of different concentrations was taken; 2.5 ml of K_3_[Fe (CN)_6_] solution (1%, w/v) and 2.5 ml of phosphoric acid buffer (pH = 6.8) were added to the test tube, mixed well, and immersed in water at 50°C for 30 min; and 2.5 ml of TCA solution (10%, w/v) was added after cooling. After 2.5 ml of supernatant was centrifuged, 2.5 ml of phosphoric acid buffer and 0.5 ml of FeCl_3_ solution (1%, w/v) were added for 10 min. Abs was determined at 700 nm with Vc as the control (40, 41).

### 2.10 Data processing and analysis

The single-factor experiment is repeated three times, using Origin 8.5 for data analysis, and the orthogonal design assistant II V3.1 is used to design and analyze the orthogonal experiment.

## 3 Results and discussion

### 3.1 Physicochemical properties of the ultrasound-assisted extraction pectin and the single acid-extracted pectin

#### 3.1.1 The yields of UAP and SAP

Using galacturonic acid as the standard substance to create a standard curve, the linear regression equation was obtained as y = 0.0131x+0.024, *R*^2^ = 0.9975, where y is the absorbance and x is the mass concentration (μG/ml). Based on the equation, the yield of the UAP is 23.98 ± 0.21%, and the yield of the SAP is 18.21 ± 0.19%. The result suggested the contribution of ultrasound-assisted treatments to pectin extraction. This is because ultrasonic waves are good at breaking down the cell walls and releasing pectin. So, the ultrasonic-assisted extraction method can improve the extraction yield of the pectin.

#### 3.1.2 Chemical composition of UAP and SAP

The composition of pectin is shown in Table 1. The UAP and the SAP all comply with the National Quality Standards of pectin in the People's Republic of China, GB 25533-2010. Compared with the SAP (71.64 ± 0.33%), the total sugar content of the UAP (78.23 ± 0.98%) is higher, which suggests the cavity, mechanical crushing, and thermolysis effects of ultrasound may destroy the cell walls of plants, causing the intracellular polysaccharides to dissolve out. The essence of pectin is a linear structure of polysaccharides, mainly composed of D-galactonic acid (2). Its galacturonic acid residues are often esterified by some groups such as methoxyl and amide. The degree of esterification is also called methoxylation, which refers to the sum of the proportions of methylesterification, acetylation, and amidation in pectin (3). Although the esterification degree of UAP (66.6%) is lower than the SAP (75%), indicating that the cavity effect of ultrasound may have broken the ester bond, resulting in the esterification degree of pectin decreasing the UAP and the SAP all belong to the high ester pectin (≥50%) (7). The change in esterification degree can affect the application field of pectin. For example, in yogurt products, high esterification degree pectin can stabilize the structure of yogurt, while adding low esterification degree pectin can prevent whey precipitation (24). The ultrasonic-assisted preparation of pectin will not affect its properties or application areas as a food additive.

**Table 1:** Test results of basic physical and chemical properties of *A. arguta* pectin.

**Quality index**	**UAP**	**SAP**	**GB 25533-2010**
Color	Light brown	Light brown	White, light yellow, light gray, or light brown
Texture	Power	Power	Power
Moisture content (%)	9.3 ± 0.2^b^	11.3 ± 0.2^a^	≤ 12
Ash content (%)	3.14 ± 0.15^b^	4.75 ± 0.12^a^	≤ 5
pH (25°C)	2.98 ± 0.22^a^	2.81 ± 0.13^a^	—
Protein (%)	0.03 ± 0.01^a^	0.03 ± 0.01^a^	—
Total sugar content (%)	78.23 ± 0.98^a^	71.64 ± 0.33^a^	—
Degree of esterification (%)	66.6	75	≥50, ≤ 50
Total galacturonic acid (%)	72.18 ± 1.57^a^	67.72 ± 1.86^b^	≥65

^a, b^Means that the data has significant differences (p < 0.05).

#### 3.1.3 Composition of monosaccharides

The monosaccharide composition of the UAP and SAP was determined by HPLC. *A. arguta* pectin is a heteropolysaccharide composed of multiple monosaccharides. As shown in Table 2, the monosaccharides and their molar ratios in UAP are Man:GlcA:Rha:GalA:Glc:Gal:Xyl:Ara:Fuc = 8.3:7.9:1.6:30:24.8:12.2:1.8:11.9:1.4. The monosaccharides and their molar ratios in SAP are Man: GlcA:Rha:GalA:Glc:Gal:Xyl:Ara:Fuc = 6.4:4.9:1.4:25.2:40.1:11.3:7.6:1.9. The results indicate that UAP will not change the type of monosaccharides, but the compositions of monosaccharides extracted from pectin by the two different extraction methods are different. The contents of GalA in the two pectins were relatively high, proving that they have better antioxidant activity (42). The content of GalA in UAp is higher than that of SAP; therefore, we predict that the antioxidant activity of UAP is stronger than that of SAP, which will be confirmed in subsequent antioxidant experiments. Pectin can be divided into four configurations, namely, homoglycan (HG), rhamnoglucuronic glycan I (RG-I), rhamnoglucuronic glycan II (RG-II), and xyloglucuronic glycan (XG) (43). In the RG-I type structure of pectin, the disaccharide unit is mainly composed of alternating rhamnose and galacturonic acid, which forms the main chain through continuous repetition; HG type and RG-II type pectin are polygalacturonic acid polymers, whose main chains are composed of α-1,4-glycosidic bond binding. The O-3 or O-4 position of the galacturonic acid residue in HG is replaced by xylose, forming XG. Visser et al. proposed a method for determining the structure of pectin molecules, where the structure is mainly RG-I type when the Rha/Gal ratio of pectin molecules is between 0.05 and 1.00; when the ratio is below 0.05, its structure is mainly HG type and RG-II type (44). The results showed that the Rha/GalA ratio of *UAP* was 0.053 and the one of SAP was 0.055, ranging from 0.05 to 1.00. However, the proportion is close to the lower limit, inferring that the molecular structure of *A. arguta* pectin belongs to a mixed structure of RG-I and HG-type domain. (Ara+GalA)/Rha reflects the ratio of RG-I branches to Rha, with a higher ratio indicating more RG-I branches. The (Ara+GalA)/Rha ratio of UAP is 15.06, and the one of SAP is 13.5, indicating that the UAP has more branched chains of RG-I than the SAP (45).

**Table 2:** Monosaccharide composition analysis of UAP and SAP.

**Sample**	**Monosaccharide composition (mol%)**										
	**Man**	**GlcA**	**Rha**	**GalA**	**Glc**	**Gal**	**Xyl**	**Ara**	**Fuc**	**Rha/ GalA**	**(Ara+Gal)/ Rha**
UAP	8.3	7.9	1.6	30.0	24.8	12.2	1.8	11.9	1.4	0.053	15.06
SAP	6.4	4.9	1.4	25.2	40.1	11.3	1.3	7.6	1.9	0.055	13.5

#### 3.1.4 Molecular weight distribution

**Figure 1:**
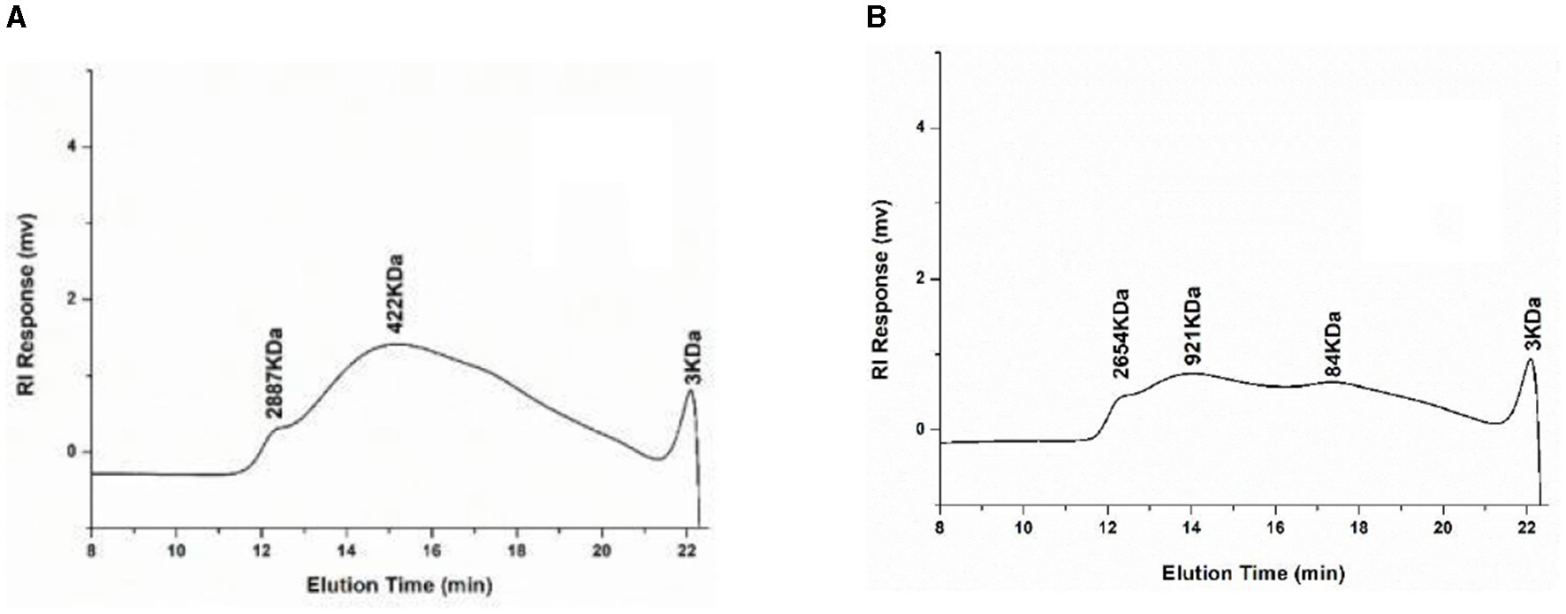
High-performance liquid chromatography of molecular weight distribution of UAP and SAP. **(A)** UAP SAMPLE; **(B)** SAP SAMPLE.

The molecular weight of pectin plays an important role in the biological activity and use of pectin (34). The standard curve equation for molecular weight is y = −0.3272+1.7945, *R*^2^ = 0.9916. Figure 1 shows the molecular weight distribution of UAP and SAP. By analyzing the molecular weight distribution, it was found that the overall distribution of pectin molecular weight in UAP and SAP has changed. The overall molecular weight distribution range of UAP is relatively concentrated, mainly distributed at 422 kDa, as shown in Figure 1A. The molecular weight distribution of SAP is relatively dispersed, with 2,654, 921, and 84 kDa, as shown in Figure 1B. This indicates that the molecular weight distribution of pectin varies with different extraction methods, which may be due to the ultrasonic pretreatment cutting off the sugar or ester chains of long-chain pectin, reducing the distribution of pectin's large molecular weight and lowering the degree of esterification, which is consistent with the results of the previous esterification degree experiment. The change in molecular weight distribution may cause changes in some physical properties of pectin (1), such as solubility and viscosity, which will be verified in the following tests.

#### 3.1.5 Fourier transform infrared analysis

**Figure 2:**
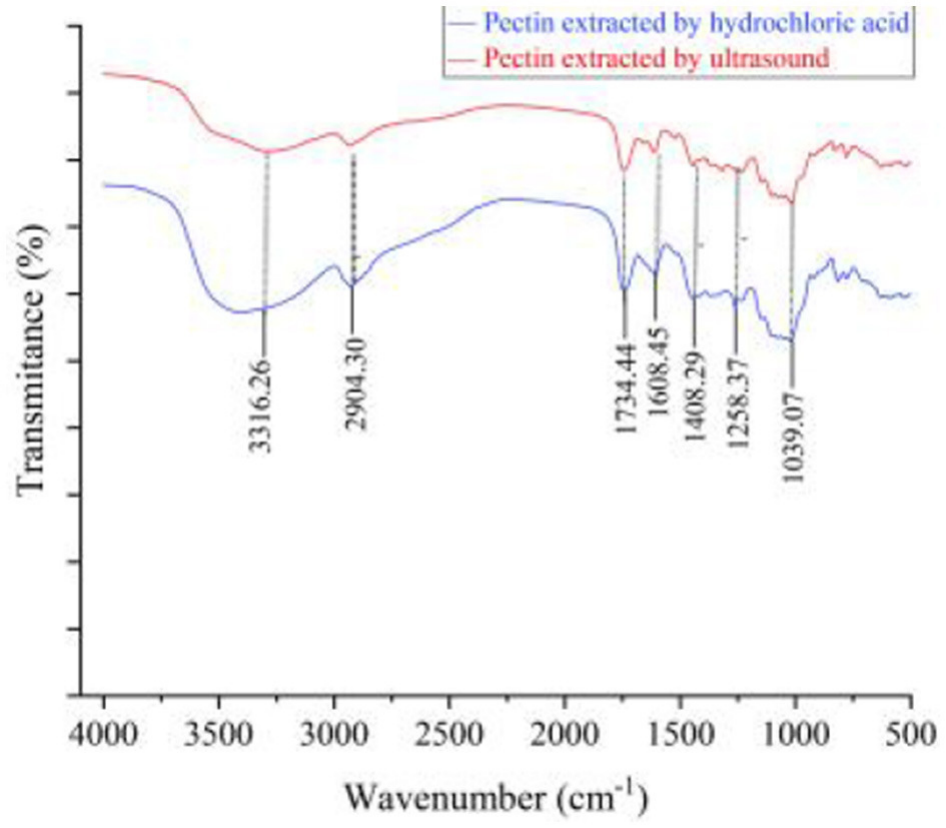
Fourier infrared spectra of UAP and SAP.

FTIR spectrum scanning scans the moisture and CO_2_ in the air as blanks and subtracts the atmospheric background from the spectrum of the sample. It can be seen from Figure 2 that pectin has a characteristic absorption peak of sugars within 500–4,000 cm^−1^. Among them, the broad absorption peak near 3,000–3,600 cm^−1^ is caused by the O-H stretching vibration on the pectin carbon ring (46). The peak between 2,795 and 2,950 cm^−1^ is caused by the C-H stretching vibration on methyl galacturonate or the carbon ring. The absorption peaks at 1,635 and 1,746 cm^−1^ are caused by carboxylate (-COO) asymmetric stretching and the C=O stretching vibration formed by carboxylate (-COOR). The peak value of UAP at the position of the carboxylic acid ester is significantly lower than the peak value of SAP, indicating that some carboxylic acid ester bonds of UAP are destroyed, so the degree of esterification is reflected, and the degree of esterification decreases. The absorption peak near 1,400 cm^−1^ is caused by the bending vibration absorption of the saturated C-H bond and the absorption peak between 1,300 and 1,000 cm^−1^ is caused by the C-O stretching vibration (1). These absorption peaks are all characteristic absorption peaks (47) unique to polysaccharide compounds, and the functional groups of pectin were not substantially changed by ultrasonic treatment.

#### 3.1.6 Scanning electron microscope imaging of UAP and SAP

**Figure 3:**
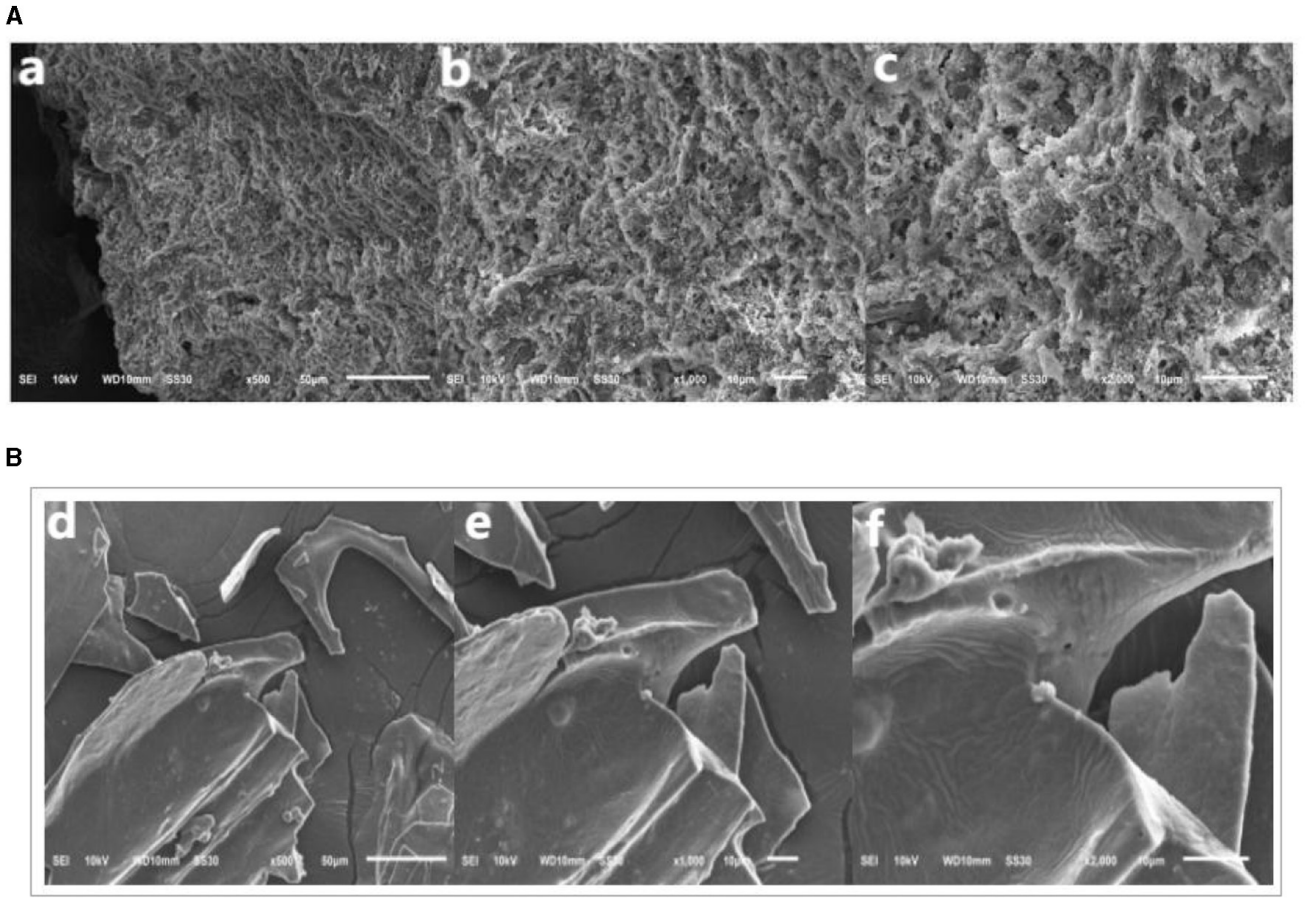
Scanning electron micrographs imaging. **(A)** UAP SAMPLE; **(B)** SAP SAMPLE.

Figure 3A shows the microstructure of UAP at 500 × , 1,000 × , and 2,000 × magnification. As shown in the figure, the surface of pectin is rough, with wrinkles and many pores. The NAP is shown in Figure 3B, which has a relatively flat and smooth surface with a sheet-like structure and small scales stacked on the surface. This is similar to the results determined by Xu et al. (46). The surface of pectin extracted by ultrasonic methods has undergone significant changes after ultrasonic treatment, resulting in porous tissue and the appearance of pores. This may be related to the changes in molecular weight distribution caused by UAP, or it may also be due to the destruction of the crosslinking between pectin molecules and the recombination of the intermolecular network structure of pectin. The surface structure of pectin prepared with ultrasonic assistance has changed, which may lead to changes in some physical properties and biological activities of pectin, which are also reflected in the following test results.

#### 3.1.7 Physical properties of UAP and SAP

As shown in Table 3, the UAP has better water retention, viscosity, and solubility than SAP. This may be due to changes in the molecular weight distribution of pectin treated with ultrasonic waves and wrinkles in its surface structure and pores, which improve pectin's physical properties such as water-holding capacity and solubility (48). This is consistent with the previous molecular weight distribution and electron microscope observation. The molecular weight and structure of the branching of pectin have important effects on its viscosity. The hydrogen bonds and hydrophobic methyl groups contained in pectin can reduce the intramolecular or intermolecular distance and increase the branching of the pectin structure, thus improving its viscosity (33). As above, ultrasonic-assisted preparation of pectin can improve the physical properties of pectin, which will improve its future application value.

**Table 3:** Test results of processing characteristics of pectin of *A. arguta*.

**Physical properties**	**UAP**	**SAP**
Water-holding capacity (g/g)	5.35 ± 0.29^a^	2.48 ± 0.15^b^
Viscosity/Pa^*^s	1.04 ± 0.08^a^	0.44 ± 0.12^b^
Hardness/N	0.68 ± 0.07^a^	0.59 ± 0.08^a^
Solubility/%	93.27 ± 1.59^a^	86.32 ± 1.22^b^

^a, b^Means that the data has significant differences (p < 0.05).

#### 3.1.8 Antioxidant activity analysis

Although ultrasonic treatment can improve the yield of pectin, pectin is a class of bioactive macromolecules. From previous experimental results, it can be seen that ultrasonic treatment can affect the esterification degree, molecular weight distribution, and physical properties of pectin. Whether this change will affect its antioxidant properties is related to its future development and application as an antioxidant food additive. Therefore, we analyzed its antioxidant properties.

**Figure 4:**
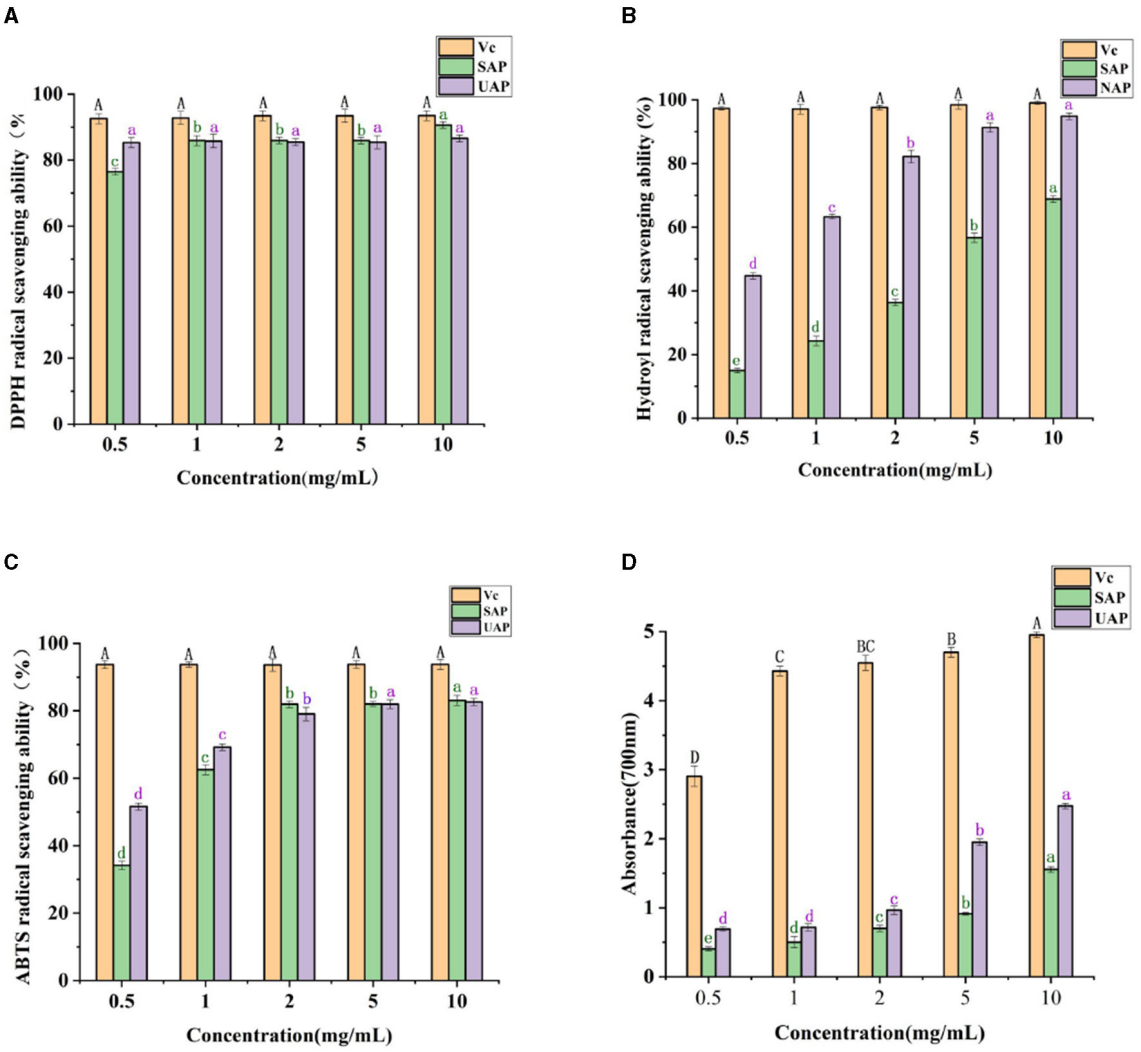
Antioxidant activity analysis of UAP and SAP. **(A)** DPPH-free radical scavenging ability of VC, UAP, and SAP. **(B)** The scavenging ability of VC, UAP, and SAP on hydroxyl radical. **(C)** The scavenging ability of VC, UAP, and SAP on ABTS-free radical. **(D)** Reducing the ability of VC, UAP, and SAP. Different superscript letters (A-D, a-d) show significant differences (Lowercase letters indicate a significant difference, *p* < 0.05; Capital letters indicate a significant difference, *p* < 0.01) within the column.

DPPH is a relatively stable free radical with a characteristic absorption peak at 517 nm wavelength (33), which is widely used to evaluate the free radical scavenging ability of antioxidant components. DPPH radical scavengers can provide hydrogen and form stable non-free radical molecules (40). Studies have shown that pectin has free radical scavenging ability, possibly due to its hydrogen donor substance, which can react with free radicals to generate more stable products and terminate free radical chain reactions (33). It can be seen from Figure 4A that in the mass concentration range of 0.5–10 mg/ml, as the mass concentration increases, the pectin shows strong DPPH-free radical scavenging ability. At the lowest concentration of 0.5 mg/ml, the DPPH-free radical scavenging ability of SAP is worse than that of UAP, and it basically remains the same as the concentration increases. When the mass concentration is 10 mg/ml, the ability of SAP is worse than that of UAP. The SAP has a stronger DPPH-free radical scavenging ability than the UAP, and the ability of Vc to scavenge hydroxyl-free radicals is always maintained at a very high level. The scavenging rate of the pectin reaches more than 50% of the scavenging rate of Vc, indicating that the *A. arguta* pectin has a strong ability to scavenge DPPH-free radicals.

The scavenging ability of OH radicals is one of the important indexes of antioxidant activity. Hydroxyl radicals can easily pass through the cell membrane, enter the cell, and react further with biological macromolecules such as carbohydrates, resulting in cell death or damage. Scavenging hydroxyl-free radicals is of great significance to organisms (41). It can be seen from Figure 4B that in the mass concentration range of 0.5–10 mg/ml, as the mass concentration increases, the ability of the pectin to scavenge hydroxyl radicals increases; when the pectin mass concentration increases from 0.5 to 10 mg/ml, the hydroxyl radical scavenging rate of UAP was significantly higher than that of SAP, indicating that ultrasonic treatment will affect the ability of the *A. arguta* pectin to scavenge hydroxyl-free radicals, which may be related to its high content of polysaccharide and galacturonic acid. The ability of Vc to scavenge hydroxyl-free radicals has always been maintained at a very high level.

ABTS+·scavenging capacity evaluation of antioxidant capacity is based on the fact that antioxidants can provide electrons or hydrogen atoms to inactivate free radicals, resulting in the change of color of ABTS+·solution (33), which is then analyzed and measured by a spectrophotometer (40). It can be seen from Figure 4C that within the mass concentration range of 0.5–10 mg/ml, as the mass concentration increases, the ability of *A. arguta* pectin to remove ABTS increases, and its ability to remove ABTS increases steadily as the concentration increases. At low concentrations, the scavenging ability of UAP is better than that of NAP, and the concentration exceeds 2 mg/ml. The difference between the UAP and SAP is not significant. The ability of Vc to scavenge hydroxyl radicals is always maintained at a very high level. When the mass concentration is 10 mg/ml, the scavenging rate of *A. arguta* pectin reaches more than 50% of the scavenging rate of Vc, indicating that *A. arguta* pectin has strong ABTS-free radical scavenging ability.

The reducing capacity of pectin is closely related to its potential antioxidant activity. Providing hydrogen atoms can break free radical chains and prevent the formation of hydrogen peroxide (33). The higher the absorbance value, the larger the sample's capacity for decrease. The reducing power of both kinds of pectin increased significantly with the increase in concentration. It can be seen from Figure 4D that in the mass concentration range of 0.5–10 mg/ml, as the mass concentration increases, the reducing power of the pectin gradually increases; when the pectin mass concentration increases from 0.5 to 10 mg/ml, the reducing ability of UAP is better than that of SAP, indicating that ultrasonic treatment improves the reducing ability of pectin; when the mass concentration is 10 mg/ml, the UAP reaches 50% of the Vc reducing ability. It shows that the UAP has the strong reducing ability. Generally speaking, the antioxidant activity of pectin is related to its structural characteristics, molecular weight, and monosaccharide composition. It has been reported that the electrophilic groups of pectin contribute to the release of H from O-H bonds, which can improve oxidation resistance. In this study, the high reducing power observed in UAP may be attributed in part to its relatively low molecular weight and high content of unmethylgalacturonic acid (43).

## 4 Conclusion

In this paper, the ultrasonic-assisted solvent method was used to prepare the pectin of *A. arguta*. Compared with the single acid extraction method, the yield of UAP (23.98 ± 0.21%) was significantly higher than that of SAP (18.21 ± 0.19%). The molecular structure of *A. arguta* pectin extracted by the ultrasound-assisted acid method belongs to a mixed structure of RG-I and HG type domain. Through structural feature analysis, the ultrasound-assisted extracted pectin (UAP) has a more branched structure than the single acid-extracted pectin (SAP). The SAP has a higher degree of esterification than the UAP. The physical property results show that the viscosity, solubility, and water-holding capacity of the UAP are better than those of the SAP. The antioxidant test results show that the hydroxyl radical scavenging and reducing powers of the UAP are superior to those of the SAP. This study provides new insight into the influence of the ultrasonic-assisted solvent method on *A. arguta* pectin and aims to provide a theoretical basis for the pharmaceutical and food industries to choose more suitable pectin extraction methods.

## Data availability statement

The original contributions presented in the study are included in the article/supplementary material, further inquiries can be directed to the corresponding authors.

## Author contributions

LL: Writing – original draft, Data curation, Methodology, Software. YS: Data curation, Software, Writing – review & editing, Resources. TW: Data curation, Resources, Visualization, Writing – review & editing. XL: Visualization, Methodology, Writing – review & editing. MS: Conceptualization, Funding acquisition, Writing – original draft. LC: Funding acquisition, Supervision, Writing – original draft.
